# Goal-directed, habitual and Pavlovian prosocial behavior

**DOI:** 10.3389/fnbeh.2015.00135

**Published:** 2015-05-27

**Authors:** Filip Gęsiarz, Molly J. Crockett

**Affiliations:** Department of Experimental Psychology, University of OxfordOxford, UK

**Keywords:** model-based, model-free, Pavlovian, reinforcement learning, dictator game, prosocial behavior, altruism, warm-glow

## Abstract

Although prosocial behaviors have been widely studied across disciplines, the mechanisms underlying them are not fully understood. Evidence from psychology, biology and economics suggests that prosocial behaviors can be driven by a variety of seemingly opposing factors: altruism or egoism, intuition or deliberation, inborn instincts or learned dispositions, and utility derived from actions or their outcomes. Here we propose a framework inspired by research on reinforcement learning and decision making that links these processes and explains characteristics of prosocial behaviors in different contexts. More specifically, we suggest that prosocial behaviors inherit features of up to three decision-making systems employed to choose between self- and other- regarding acts: a goal-directed system that selects actions based on their predicted consequences, a habitual system that selects actions based on their reinforcement history, and a Pavlovian system that emits reflexive responses based on evolutionarily prescribed priors. This framework, initially described in the field of cognitive neuroscience and machine learning, provides insight into the potential neural circuits and computations shaping prosocial behaviors. Furthermore, it identifies specific conditions in which each of these three systems should dominate and promote other- or self- regarding behavior.

The existence of prosocial behaviors—actions that increase the welfare of others, often at cost to oneself—remains an enduring scientific puzzle. At a first glance such behaviors are inconsistent with the axiom of rational self-interest in neo-classical economics, the law of natural selection in evolutionary biology and the law of effect in behavioral psychology. Nevertheless, prosocial behaviors are widespread across cultures and also found in the animal kingdom (Waal, 1997; Henrich et al., 2001; Engel, 2011). One persisting set of questions concerns the extent to which such behaviors are guided by an “altruistic” motivation to improve the welfare of others. For decades, scientists have debated whether altruistic motivation even exists, and if so, whether it is “rational” in the sense of satisfying real preferences, or rather is a by-product of our evolutionary history. We suggest that to answer both of these questions it is necessary to examine different motivations, and the prosocial behaviors they give rise to, in terms of their underlying cognitive and neural mechanisms.

Here we will show that many theories about the causes of prosocial behaviors can be organized and integrated under a reinforcement learning and decision-making (RLDM) framework, initially developed in the field of cognitive neuroscience and machine learning (Sutton and Barto, 1998; Daw et al., 2005; Dayan, 2008; Dolan and Dayan, 2013). We will argue that this scheme not only streamlines the seemingly heterogeneous landscape of motivations driving prosocial behaviors, but also provides insight into the mechanisms governing them. In a broader context, this proposition also complements recent suggestions that an RLDM framework can help explain patterns of moral judgments (Crockett, 2013; Cushman, 2013) and elucidate computations underlying social cognition (Dunne and O’Doherty, 2013).

As prosocial behaviors can be expressed in many ways and describing them all is beyond the scope of this paper, we will focus here on sharing, consoling, helping and cooperating. To tackle the problem more formally, we will attempt, where possible, to use examples from game theory—most notably the *Dictator Game*, in which a participant receives a certain endowment and must decide whether to transfer some portion of it to another participant (Forsythe et al., 1994). We will start our considerations with a brief outline of the RLDM framework and its underlying computations. Subsequently, we will consider how three decision systems described by it, either in isolation or through interacting with one another, can give rise to different characteristics of prosocial behavior.

## The RLDM Framework

The RLDM framework addresses the problem of how artificial agents should make choices and learn from interactions with the environment to achieve some goal (Sutton and Barto, 1998). It was built on the Markov decision processes framework, according to which every decision-making problem can be decomposed into four elements: the agent’s situation (state), which defines currently available outcomes; the agent’s choices (actions), which define currently available behaviors; the agent’s goal (reward function), which defines how rewarding given outcomes are, and finally the model of the environment (transition function), which defines how given choices lead to certain situations (Sutton and Barto, 1998; van Otterlo and Wiering, 2012). This formalization has been used in three classes of algorithms aiming to optimize decision-making: *model-based planning*, which infers the best decisions from knowledge of the environment; *model-free learning*, which learns the best decisions from the outcomes of past actions; and *a priori programming*, which defines the best decisions for each situation beforehand, for example on the basis of performed simulations (Sutton and Barto, 1998; van Otterlo and Wiering, 2012).

Reinforcement learning algorithms, in principle, can be employed in any domain, and each of them, given enough information, can prescribe an optimal policy for a wide range of problems. One could speculate that such universal tools would be advantageous for any organism struggling for survival and therefore their emergence should be promoted by evolution. Indeed, a large body of evidence suggests that similar algorithms are present in the mammalian brain and are embedded in the *goal-directed*, *habitual*, and *Pavlovian* decision-making systems (Daw et al., 2005; Dayan, 2008; Rangel et al., 2008; Balleine and O’Doherty, 2010; Dolan and Dayan, 2013).

**Figure 1:**
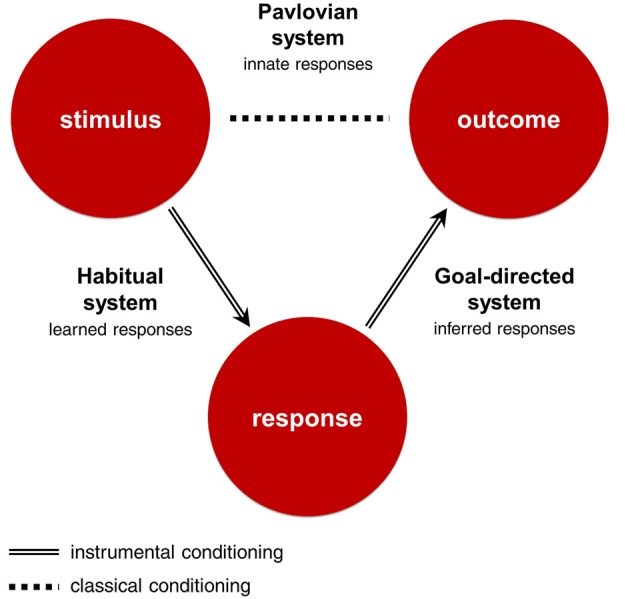
**Stimulus-Response-Outcome contingency and corresponding decision-making systems**. The Stimulus-Response-Outcome association is learned through mechanisms of instrumental conditioning, and the Stimulus-Outcome association through mechanisms of classical conditioning. The goal-directed system uses response-outcome associations to infer which actions will bring the best outcomes from the perspective of current goals. The habitual system uses stimulus-response associations to emit responses that produced the best outcomes in similar situations in the past. The Pavlovian system emits innate responses to outcomes that were significant in our evolutionary history or stimuli that were associated with these outcomes.

All three RLDM systems learn about some part of the stimulus-response-outcome contingency, and use this knowledge to make decisions (Figure 1; Table 1). The *goal-directed system* uses response-outcome associations to infer which responses will bring the best outcomes from the perspective of current goals. It can be characterized as deliberate, dominating at the beginning of learning, dependent on working memory and sensitive to sudden changes in motivational states. The *habitual system* uses stimulus-response associations to emit responses that produced the best outcomes in similar situations in the past. It dominates in later stages of learning, is independent from working memory and insensitive to sudden changes in motivational states. These two systems are called ‘instrumental’ as they use associations learned through actions. In contrast, the *Pavlovian system* emits reflexive responses to outcomes that were significant in our evolutionary history or stimuli that were associated with these outcomes through the mechanisms of classical conditioning. For example, pavlovian system can emit approach reaction to stimuli associated with food and withdrawal reaction to stimuli associated with pain. Importantly, these responses might be highly sophisticated and sensitive to contextual cues, as in the case of a flight reaction to distal threat and a fight reaction to proximal threat (McNaughton and Corr, 2004). Pavlovian responses, unlike those of the instrumental systems, are inborn, inflexible and preprogrammed by evolution. As such, this system is unable to update its responses when they produce undesirable outcomes. Instead, Pavlovian responses are beholden to the evolutionary context in which they evolved. As a result, Pavlovian responses are efficient solutions to a range of situations that were important in our phylogeny, but may sometimes produce counterproductive behaviors when the current environment demands a more tailored response.

**Table 1:** **Properties of three decision-making systems**.

Goal-directed system	Habitual system	Pavlovian system
• Employs model-based planning algorithms	• Employs model-free learning algorithms	• Employs a priori programmed solutions
• Deliberate	• Automatic/Learned	• Automatic/Inborn
• Dominating at the beginning of learning	• Dominating in late stages of learning	• Can dominate at all stages of learning
• Dependent on working-memory	• Independent from working-memory	• Independent from working-memory
• Sensitive to sudden changes in motivational states	• Insensitive to sudden changes in motivational states	• Sensitive to sudden changes in motivational states
• Sensitive to consequences of actions	• Insensitive to consequences of actions	• Insensitive to consequences of actions

The RLDM framework shares many similarities with dual-process accounts of judgment and decision making, in which one system is usually described as emotional, intuitive, domain-specific and automatic, and a second system as cognitive, reflective, domain-general and controlled (Stanovich and West, 1998; Evans, 2008). However, neither of these systems can be directly mapped to the RLDM framework because of a few important differences. First of all, the RLDM systems do not distinguish between “emotion” and “cognition”; rather, all of the RLDM systems rely on emotions, in the sense of processing the affective valence of events. Furthermore, the RLDM systems use well-specified algorithms that do not have an equivalent in dual-process frameworks. Finally, the RLDM framework emphasizes a distinction between inferred, learned and inborn responses—one that is often overlooked by other frameworks. Therefore, it can be concluded that, despite some overlap, the RLDM framework is distinct from traditional dual-process accounts in psychology. In the following sections, we will describe the computational properties and neural substrates of the goal-directed, habitual and Pavlovian systems, as well as procedures used to differentiate between them.

### The Goal-Directed System

Model-based planning algorithms select the best decision on the basis of available information—extracted, for instance, from task instructions (Daw, 2012). The *tree-search* algorithm is one of the main examples of this approach. It utilizes a model of the environment to simulate the outcomes of each possible sequence of actions and then evaluates the cumulative value of them in the light of current goals (Daw et al., 2005; Daw, 2012). By considering each possible scenario, this approach ensures making an optimal decision. However, it has some limitations. The first problem is that the agent might not have enough information about the environment to foresee the consequences of each action. Computer scientists deal with this issue by adding a component to the above algorithm that infers the unknown contingencies (Littman, unpublished doctoral dissertation). The second problem is intractability—the more potential sequences of actions and the more complex relationships between them, the more probable it is that the agent will not have enough time and computational power to evaluate all possible outcomes. To prevent this, model-based algorithms use heuristics to narrow down the extent of considered scenarios (Daw, 2012). Other approaches propose that model-based planning, rather than investigating the consequences of each action, could also start with the desirable end state and try to infer, for example through a procedure known as Bayesian model inversion, the actions that could lead to this state (Botvinick and Toussaint, 2012; Solway and Botvinick, 2012).

Employing tree-search and Bayesian model inversion algorithms to behavioral tasks shows that these algorithms share many characteristics of the goal-directed system, including sensitivity to changing circumstances and having an advantage at the beginning of learning over other RLDM systems (Daw et al., 2005; Keramati et al., 2011; Solway and Botvinick, 2012). Perhaps the most surprising common feature of model-based algorithms and the goal-directed system is the slow pace of operation. Serial processing of standard computer processors greatly limits how many sequences of actions can be evaluated in a unit of time. In the brain, which mostly relies on parallel processing (Alexander and Crutcher, 1990), this problem should be much less pronounced. However, taxing participants’ working memory with a demanding task impairs functioning of the goal-directed system (Otto et al., 2013), and suggests that, at least in part, the goal-directed system also employs serial processing (Zylberberg et al., 2011).

Although many brain regions underlie the goal-directed system, the dorsolateral prefrontal cortex (DLPFC) stands out as one of its main neural substrates. First, fMRI studies show that the DLPFC is engaged in tasks involving cognitive processes related to model-based computations, such as: forward planning (Kaller et al., 2011; Wunderlich et al., 2012), organizing working memory content (Owen et al., 2005) and updating a model of the environment (Gläscher et al., 2010). Second, single-unit recordings in monkeys’ DLPFC show that neurons in this region encode all variables crucial for performing tree-search and Bayesian model inversion algorithms—namely the potential outcomes, actions and goals (Abe and Lee, 2011; Genovesio et al., 2014). Third, modeling of DLPFC activity suggests that it shows some characteristics of serial processing (Yildiz and Beste, 2014). Finally, disrupting DLPFC function using TMS impairs participants’ performance in tasks requiring model-based computations (Smittenaar et al., 2013).

The orbitofrontal cortex and anterior caudate nucleus have also been identified as important components of the goal-directed system (Balleine and O’Doherty, 2010; Gläscher et al., 2010). However, recent evidence suggests that these regions might integrate information from all three decision-making systems (Daw et al., 2011; Liljeholm and O’Doherty, 2012; Wunderlich et al., 2012; Lee et al., 2014). For these reasons in further sections we will concentrate on DLPFC and treat its activation as being consistent with an involvement of the goal-directed system, although we note this assumption should be treated with caution as it represents reverse inference—i.e., the logical fallacy of inferring the involvement of a particular cognitive function from brain region activation, when this region is not engaged exclusively by this cognitive function (Poldrack, 2006; but see: Hutzler, 2014).

### The Habitual System

Model-free learning algorithms ignore the model of the environment and instead integrate the history of consequences of a given action into a *cached* *action value* (Sutton and Barto, 1998; Dayan, 2008). Although there are many different procedures describing this process, here we will focus on the family of actor-critic models that have been inspired by neuroscience (Joel et al., 2002). In essence, in these models the *actor* automatically choses the action with the highest expected value. The *critic*, in turn, evaluates the outcomes of this action and “teaches” the actor how to update the expected value—by adding to the previous expectation a fraction of a *prediction error* (the difference between the actual and expected value). As this algorithm relies on a single cached value, refined incrementally, it is much more computationally efficient than its model-based alternative.

Efficiency of the model-free algorithms comes at a cost: as model-free algorithms require extensive experience to optimize their policies, they are outcompeted by model-based algorithms when rapid changes in the environment invalidate what has been learned so far (Carmel and Markovitch, 1998). This property is related to the insensitivity of the habitual system to sudden changes in motivational states and the gradual transition from goal-directed to habitual control with experience. Both of these features are well illustrated by the example of the devaluation procedure (Gottfried et al., 2003). In this procedure rats are first trained to make an action (such as pressing a lever) to obtain a rewarding outcome, e.g., sweetened water. At some point, the value of water is artificially diminished (i.e., devalued) by pairing it with nausea-inducing chemicals, which makes the previously desired outcome aversive. If the devaluation procedure is carried out early in training, when the habit of pressing the lever is still weak, rats will not perform the action that delivers the now-devalued sweetened water in this situation. But if the devaluation procedure is employed after extensive training, rats will keep pressing the lever in this situation, even though they are no longer interested in the outcome of this action.

Neuroscientific evidence in general supports the actor-critic model as a plausible computational approximation of the habitual system, although details of how it is actually implemented in the brain are still under debate (Dayan and Balleine, 2002; Joel et al., 2002). First, the division between the actor and the critic is mimicked by the dissociation between the action-related dorsal and reward-related ventral parts of the striatum (O’Doherty et al., 2004; FitzGerald et al., 2012). Furthermore, responses of neurons in both of these regions resemble prediction errors (Schultz, 1998; Joel et al., 2002; Stalnaker et al., 2012). Finally, parallel processing in the striatum (Alexander and Crutcher, 1990; Yildiz and Beste, 2014), its dense connections with sensorimotor cortex (Ashby et al., 2010) and increasing involvement of its dorsal part with training (Tricomi et al., 2009) explains the fast responses of the habitual system, in comparison to its goal-directed counterpart.

### The Pavlovian System

Instead of letting an algorithm infer or learn the best policy, one can simply program *a priori* the best action for any given situation and execute it automatically whenever this situation is encountered (van Otterlo and Wiering, 2012). This could be done either on the basis of the programmer’s knowledge or algorithmically, for example using the Monte Carlo method, which identifies the best responses by simulating random action sequences in a given environment and averages the value of outcomes for each response in a given situation (Sutton and Barto, 1998). The main shortcomings of this strategy are its specificity and inflexibility. As the variety of situations in the real world is potentially infinite, it is unfeasible to pre-program appropriate responses to all of them, and therefore one has to focus on some subset of events. One solution to this problem is to generalize rules defining when the given action should be executed. However, such generalizations increase the risk of encountering exceptions to the rule, where the triggered action is inappropriate in the given context.

The Pavlovian system bears many similarities to this strategy, as it reflexively executes unconditional approach and withdrawal responses to classes of stimuli that were important in our evolutionary history. Characteristics of cues triggering these unconditional responses were probably determined by relative costs of omissions vs. false alarms in our phylogeny (Schmajuk, 1987; Parker and Smith, 1990). For example, it might have been more adaptive to overgeneralize features triggering reactions to potential threats, as in the case of a startle response induced by suddenness, because omissions could result in death, whereas false alarms merely cost energy. Classical conditioning can be thought of as a mechanism that helps the organism to generalize inborn Pavlovian responses to situations not consistently paired with unconditional stimuli in our evolutionary history, but nevertheless predicting their occurrence in the current environment.

Inflexibility of Pavlovian responses can have maladaptive consequences in certain contexts—classically illustrated in the negative auto-maintenance procedure (Williams and Williams, 1969). In the first phase of this procedure, food is reliably paired with a conditioned stimulus, until the animal starts to approach not only the food, but also the conditioned stimulus. In the second phase, food is delivered only if the animal refrains from approaching the conditioned stimulus. Although in this context approach behaviors bring negative consequences, animals will repeat them for thousands of trials without obtaining any reward (Killeen, 2003). This procedure not only demonstrates the rigidity of the Pavlovian system, but also its strength when it is pitted against the two other systems (Dayan et al., 2006).

The Pavlovian system has many similarities with the habitual system, and in many cases their influences might be hard to distinguish, as both systems involve responses that are automatic, independent of working memory and insensitive to the predicted consequences of actions (Table 1). To prove that a response has a Pavlovian rather than a habitual character one has to show that it is inborn, instead of learned. Furthermore, Pavlovian responses are sensitive to the current motivational state of the organism, in contrast to habitual responses (Dayan and Berridge, 2014). Specifically, conditioned stimuli associated with a particular outcome will trigger automatic approach reactions only if the animal is currently in a state in which the associated outcome is rewarding. For example, rats are typically attracted to a lever associated with sucrose solution delivery and repulsed by a lever associated with a highly saline solution delivery (Robinson and Berridge, 2013). However, rats that are injected with a drug mimicking the state of salt deprivation start to express Pavlovian approach responses towards the lever previously associated with saline solution, such as sniffing, grasping and nibbling. These reactions occur even though approaching the lever does not deliver any outcome and therefore has no instrumental value or even has a negative action value because it was previously associated with an aversive outcome.

Importantly, the Pavlovian system can invigorate or inhibit the responses of the instrumental systems—a phenomon known as Pavlovian-to-instrumental transfer (PIT; Talmi et al., 2008; Lewis et al., 2013). Specifically, the presence of appetitive stimuli has been shown in many experiments to invigorate instrumental approach reactions and inhibit instrumental withdrawal reactions (Talmi et al., 2008; Corbit and Balleine, 2011; Huys et al., 2011; Guitart-Masip et al., 2014). For example, Huys et al. (2011) have shown that visual cues previously associated with monetary rewards speeded movement towards the target stimulus, and slowed movement away from the target stimulus. In contrast, visual cues previously associated with monetary losses have been shown to inhibit instrumental approach reactions and invigorate instrumental withdrawal reactions (Huys et al., 2011; Lewis et al., 2013). The precise mechanisms underlying PIT are still not well understood. It has been proposed that PIT could modulate instrumental approach and withdrawal reactions either through increasing the expectation of a specific outcome or increasing positive and negative arousal (Corbit and Balleine, 2005, 2011).

At the neural level, the most important substrates of the Pavlovian system are the amygdala, which is crucial for acquiring associations between conditioned and unconditioned stimuli (Savage and Ramos, 2009), and the ventral striatum, which takes part in processing the value of primary rewards and punishments, as well as the value of conditioned stimuli (Liljeholm and O’Doherty, 2012). Both of these structures also play a crucial role in PIT (Corbit and Balleine, 2005, 2011; Talmi et al., 2008; Lewis et al., 2013). At the level of neurotransmitters, Pavlovian approach reactions have been predominantly associated with dopamine and Pavlovian inhibition with serotonin (Boureau and Dayan, 2011; Crockett et al., 2012; Guitart-Masip et al., 2014).

## An RLDM Framework for Prosocial Behavior

Having characterized the three RLDM systems in more detail, it is important to ask why the RLDM framework is suitable for describing and explaining prosocial behaviors. It could be argued that choice between other- and self-regarding acts is just an ordinary decision-making problem for the brain, and therefore it should be resolved by general-purpose decision-making systems. In this scenario, processes underlying prosocial behaviors would face the same challenges as any other decision and in consequence inherit the exact characteristics of whichever system is primarily responsible for them.

An alternative perspective suggests that, due to the importance of social interactions for human survival, selective pressures could have produced dedicated brain circuits responsible for other-regarding acts, such that they could be motivated by unique processes extending beyond reinforcement learning mechanisms (Field, 2004). We do not exclude this possibility; however we argue that a strong separation between decision-making systems and circuits responsible for prosocial behaviors is unlikely in light of the substantial overlap between social and economic decisions on the neural and behavioral level (Ruff and Fehr, 2014). Following the debate about *common currency* in neuroeconomics—according to which the brain makes choices using a single scale that represents the values of options irrespective of the social or non-social nature of stimuli (Levy and Glimcher, 2012; Ruff and Fehr, 2014)—we suggest instead that brain circuits specialized for prosocial behaviors, if such circuits exist, could either be embedded within the general-purpose RLDM systems or constitute an input and output for them.

In the following sections, we will review evidence showing that many instances of other-regarding acts resemble goal-directed, habitual or Pavlovian decisions. Furthermore, we will suggest in what contexts each of these systems should promote or suppress prosocial behaviors from the perspective of reinforcement learning. Future work will need to address to what extent this framework is sufficient to explain the broad array of observed patterns of prosocial behavior and to what extent it needs to be supplemented by other mechanisms.

### Goal-Directed Prosocial Behavior

A desire to achieve some goal, through the means of other-regarding acts, is perhaps the most straightforward motivation driving prosocial behaviors. Evolutionary biologists and neo-classical economists proposed that the superordinate goal of all behaviors is to propagate one’s own genes and maximize one’s own utility, respectively (Hamilton, 1964; Hollander, 1977). Consequently, according to these perspectives, all other-regarding acts are ultimately selfish. Alternative accounts proposed that some people might have genuine preferences for others’ welfare or act in accordance with moral principles (Batson, 1994; Fehr and Fischbacher, 2002). In this section we will review how self-interest can motivate prosocial behavior and show that to appreciate the benefits of other-regarding acts, people must simulate the short- and long-term consequences of their behavior on the basis of knowledge about the environment—an ability constituting a hallmark of the goal-directed system, requiring model-based computations and likely implemented by the DLPFC. Furthermore, we will suggest that the same mechanisms are employed in the pursuit of non-egoistic goals.

The first mechanism through which self-interest can motivate prosocial behavior is direct reciprocity, where helping someone increases the likelihood that they will return the favor (Trivers, 1971). Direct reciprocity has been mostly studied using the repeated prisoner’s dilemma, in which two players have to decide whether to cooperate or defect (Rapoport, 1965). If both cooperate, each gets a moderate reward; if both defect, each gets only a small reward. If one defects while the other cooperates, the defector gets a large reward while the cooperator gets nothing.

If the game is played only once, from the perspective of an individual it is always better to defect, because this either exploits the other’s cooperativeness or avoids exploitation of the individual. If the game is repeated, however, in the long run mutual cooperation maximizes the outcomes of both players. Therefore, each player has to establish when cooperative moves have a chance of being reciprocated and adjust their strategy accordingly. The most successful strategies (“tit-for-tat”) always start with cooperative move and copy responses of the opponent from the previous encounter thereafter (Axelrod and Hamilton, 1981). Furthermore optimal strategy should be also sensitive to the probability of future interactions and switch from the above “tit for tat” behavior to “always defect” when this probability is low (Rand and Nowak, 2013).

Direct reciprocity is common in humans but surprisingly rare in other animals (Clutton-Brock, 2009). One reason for this might be that it requires sophisticated cognitive abilities absent in simpler organisms (Stevens and Hauser, 2004). A well-developed goal-directed system might be one such ability. In the repeated prisoner’s dilemma an agent has to resolve a conflict between smaller rewards now, resulting from defection, and cumulatively larger rewards later, resulting from long-term cooperation—a task reportedly hard for animals (Green et al., 1995). The goal-directed system has the capacity to promote optimal strategies for the current situation as it is able to evaluate the cumulative value of outcomes of different action sequences and override automatic responses. Therefore it can choose a tit-for-tat strategy when the probability of future interactions is high, but switch to defection when it is low—a pattern often observed in behavioral experiments (Bó, 2005; Rand and Nowak, 2013). Consistent with the involvement of the goal-directed system in direct reciprocity, holding a belief that one’s interaction partner will reciprocate in an iterated prisoner’s dilemma, relative to lacking insight into the partner’s strategy, is associated with greater activity in the DLPFC (Sakaiya et al., 2013). The same brain region was shown to be engaged in a prisoner’s dilemma by prosocial individuals when they decided to defect, as well as in antisocial individuals when they decided to cooperate, suggesting that it might be involved in goal-directed adjustments of dominant behaviors (Rilling et al., 2007).

Another mechanism through which self-interest could motivate prosocial behavior is indirect reciprocity—that is, gaining personal benefits from having a good reputation (Nowak and Sigmund, 2005). Laboratory experiments show that being publicly generous pays back, as third parties tend to reward those who are kind to others (Wedekind and Braithwaite, 2002; Servátka, 2010). Behaving in line with social norms also improves one’s public image (Andreoni and Bernheim, 2009; Bereczkei et al., 2010) and being altruistic increases one’s sexual attractiveness (Farrelly et al., 2007; Barclay, 2010). Perhaps the strongest evidence that people are in fact driven by such motivations comes from the studies that eliminate the opportunity to improve one’s reputation by making all prosocial acts anonymous, which greatly decreases the willingness to share an endowment (Bereczkei et al., 2010; Franzen and Pointner, 2012; but see: Barmettler et al., 2012). Importantly, prosocial behaviors are performed more vigorously in public only if they signal to the audience intrinsic prosocial motivations; this vigor is diminished if the person could appear to be acting prosocially to obtain external rewards (Ariely et al., 2009). Differential prosocial behavior between public and private conditions can already be observed in 5-year-olds (Engelmann et al., 2012; Leimgruber et al., 2012). Moreover, this effect is sensitive to the features of the observer: 5-year-olds share more resources when the person looking can potentially reward them for good deeds, in comparison to the situation when they cannot, suggesting that this behavior is, at least in part, deliberate and strategic (Engelmann et al., 2013). Such reputation management probably depends on the development of theory of mind, understood as an ability to attribute mental states to others, as it enables individuals to judge how their actions will be evaluated by others. Consistent with this, chimpanzees and children with autism, both characterized by an underdeveloped theory of mind, do not seem to be concerned about their own reputation (Izuma et al., 2011; Engelmann et al., 2012). On the other hand, studies investigating influence of individual differences in theory of mind on prosocial behaviors found mixed results (Edele et al., 2013; Artinger et al., 2014).

How are concerns about one’s reputation incorporated into prosocial decisions? We speculate that the goal-directed system treats others’ minds as a part of the environment and simulates their contents in order to determine the consequences of one’s actions for one’s own reputation. In line with this idea, some studies suggest that in economic games the engagement of theory of mind is related to activity in the DLPFC, among other areas (Yoshida et al., 2010), and involves computations similar to tree-search (Yoshida et al., 2008) and Bayesian model inversion algorithms (Baker et al., 2009; Moutoussis et al., 2014). Consistently, disruption of DLPFC by TMS impairs the accuracy of theory of mind (Costa et al., 2008) and diminishes concerns about one’s reputation (Knoch et al., 2009).

Avoiding punishments for violating social norms is another factor motivating people to behave prosocially. This problem has been studied using the ultimatum game, in which a proposer decides how to divide a sum of money (e.g., $10) between themselves and another participant, similarly to the dictator game (Güth et al., 1982). However, unlike in the dictator game, the recipient can reject the offer and then both of the participants get nothing. Recipients often reject offers perceived to be unfair—a behavior interpreted as costly punishment of a fairness norm violation (Oosterbeek et al., 2004; Henrich et al., 2006) or, in the case of some individuals, as spite (Brañas-Garza et al., 2014). Perhaps anticipating this, proposers usually share money to a higher degree than in the standard dictator game. On the other hand, reducing the negative consequences of offer rejection for the proposer proportionally decreases their offers (Handgraaf et al., 2008). Together these findings suggest that there is a strategic component to proposers’ prosocial behavior.

Children playing the role of proposer in the ultimatum game dramatically increase their offers as soon as they are able to pass the false-belief task, indicating the development of a theory of mind (Sally and Hill, 2006; Castelli et al., 2010; Takagishi et al., 2010). However, it is not clear if this developmental milestone increases a preference for fairness or the ability to strategically adjust fair behavior to benefit oneself. To extract the purely strategic component of prosocial behavior, researchers have compared offers in the dictator game and the ultimatum game made by the same individual. The difference in offers between these two games rises substantially from early to middle childhood (Sally and Hill, 2006) and is associated with maturation of the DLPFC (Steinbeis et al., 2012), suggesting involvement of the goal-directed system. Consistent with this, prosocial behavior in the ultimatum game, relative to the dictator game, is associated with stronger activation in the right DLPFC, lateral OFC and caudate nucleus (Spitzer et al., 2007). Furthermore, stimulation of the right DLPFC by tDCS not only increases donations in the ultimatum game, but also decreases donations in the dictator game (Ruff et al., 2013), suggesting that the right DLPFC plays a causal role in calculations aiming to maximize personal benefits.

If the norm of fairness was the only factor regulating how much people share in ultimatum games, people should never give more than half of their initial endowment. In reality, however, some subjects do (Chang et al., 2011), which implies the existence of additional motives. It can be argued that compliance with others’ expectations, rather than compliance with cultural norms, might be a more sensitive strategy for building a good reputation and avoiding punishments. Consistent with this, in a study by Chang et al. (2011) participants playing the ultimatum game tried to make offers meeting the expectations of the other person, rather than splitting the endowment evenly. Moreover, this behavior was related to activation in the DLPFC, among other regions.

So far we have reviewed studies suggesting that prosocial actions are motivated at least in part by strategic self-interest and likely fall within the purview of a goal-directed RLDM system. Nevertheless, there is also evidence that even in the absence of personal incentives to behave prosocially, some people are still willing to help others (Batson et al., 1999; Franzen and Pointner, 2012). As the goal-directed system enables the pursuit of any goal, one potential explanation for these selfless behaviors is that some people are simply motivated to act in accordance with moral principles.

Several different types of moral values inform human social behavior and there is an ongoing debate about which ones can be considered universal (Haidt, 2007). In the context of sharing, three values seem to be particularly important: equality, meritocracy and effectiveness (Charness and Rabin, 2002; Fong, 2007; Konow, 2010). People seem to incorporate these values into decisions to share resources, giving more money to the less fortunate, those who deserve it and those for whom the transfers are more effective, respectively (Brañas-Garza, 2006; Dawes et al., 2007; Hsu et al., 2008; Almås et al., 2010). Moreover, some people reject offers favoring themselves over the other person (Blake and McAuliffe, 2011), are more willing to donate money to charities than to students (Konow, 2010) and are willing to pay money to ensure the implementation of the most effective charity option (Null, 2011). Although these studies do not exclude an involvement of egoistic motivations, they clearly show that people are concerned about the consequences of their actions for other people from the perspective of moral principles.

### Habitual Prosocial Behavior

Previous work combining the RLDM framework with game theory has demonstrated that simple model-free algorithms, which gradually increase the probability of successful actions and decrease the probability of unsuccessful actions, better describe human behavior than *a priori* programmed optimal strategies in a variety of two-player non-cooperative economic games (Erev and Roth, 1998; Sarin and Vahid, 2001). However, without making any additional assumptions, these same model-free algorithms predict a decrease in cooperation over time in a repeated prisoner’s dilemma, in sharp contrast to observed human behavior, which is characterized by an increasing tendency to cooperate over time (Erev and Roth, 2001). Computer simulations suggest that model-free algorithms are able to learn to cooperate in a variety of cooperative games under the assumption that outcomes of cooperation are satisfactory for both partners of interaction, and are guaranteed to do so if in addition cooperation is more satisfactory than actions maximizing one’s own payoffs at the cost of the other player (Sarin, 1999; Macy and Flache, 2002). What mechanism could ensure that cooperation is satisfactory for both players and more satisfactory than the maximizing option? Social norms of reciprocity and fairness, creating additional utility from acting according to these norms, could be one possibility (Fehr and Schmidt, 1999). Alternatively, but not exclusively, the goal-directed system could interact with the habitual system and reinforce prosocial actions which fulfill some goals, for example actions that boost one’s reputation or are in line with some moral values. Consistently, such actions are often found to be associated with increased activity in ventral and dorsal striatum (Hsu et al., 2008; Izuma et al., 2010; Tricomi et al., 2010). According to the RLDM framework, frequent rewarding of a given action should lead to a gradual transition from goal-directed to habitual control of that action (Daw et al., 2005). Consequently, with extensive experience, certain actions can become automated and valued in and of themselves, irrespective of their consequences. In the following, we will show that many reported observations of prosocial behaviors suggest that these behaviors have features of habits and intrinsically valued actions.

The notion of prosocial habits is similar to the social heuristics hypothesis, according to which other-regarding acts in one-shot anonymous games stem from intuitive processes, shaped by successful strategies in social interactions and internalization of cultural norms (Rand et al., 2014). In line with both accounts, playing a repeated prisoner’s dilemma, in which the payoff structure promoted cooperation, was shown to increase other-regarding behavior in a subsequent battery of one-shot economic games, in comparison to a condition where the payoff structure promoted defection (Peysakhovich and Rand, 2013). It is important to note, however, that interpreting this result as evidence for habit acquisition requires making a few assumptions, as in the classic RLDM literature habits are usually tied to a specific situation and action, rather than a general behavioral tendency expressed across different contexts. Although the generalization of actions across situations has been observed in the case of motor habits (Krakauer et al., 2006; Hilario et al., 2012), it is very limited in scope. Therefore, future studies need to clarify if prosocial habits can spill over into novel situations and generalize to similar actions to a much greater extent than motor plans, or if these findings can be explained by other phenomena.

The possibility that some prosocial actions might be habitual and chosen without regard for their consequences fits many findings in behavioral economics. According to public goods theory, if rational individuals were interested in achieving some desirable state of the social environment, then government spending on that cause should diminish their willingness to financially support it, an assumption known as the “crowding out” hypothesis (Steinberg, 1991; Andreoni, 1993). Indeed, some experiments demonstrated this effect by forcefully taking money from a participant’s endowment and, in a transparent manner, transferring it to a given cause (Eckel et al., 2005). However, other experimental and field studies, using slightly different procedures, found incomplete crowding out (Andreoni, 1993; Ribar and Wilhelm, 2002). Similarly, satisfying the norm of fairness in a dictator games by providing an equal endowment to the dictator and the recipient does not completely diminish dictators’ willingness to share the endowment (Konow, 2010; Korenok et al., 2013). Furthermore, although people are eager to donate money to charities, most are unwilling to spent money to learn which actions support charities efficiently (Null, 2011). These studies suggest that the aid itself is not the main purpose of these acts.

In line with this, some people are willing to donate money to charity even if they know that their actions are completely ineffective. For example, Crumpler and Grossman (2008) introduced a variation of the dictator game in which any action of the dictator was counterbalanced by the experimenter, such that a donation of $10 to charity would result in the experimenter donating nothing, whereas a donation of $0 would result in the experimenter donating $10. Nevertheless, subjects still gave away about 20% of their endowment in this situation, although there was no reason to do so if they were solely motivated by concern about the welfare of the charity. This effect was still present, albeit smaller, when researchers controlled for the concern about the welfare of the experimenter (Tonin and Vlassopoulos, 2014). It is unlikely that the above findings were driven by the expectations of the experimenter as the procedure was double-blind and also because other studies examining dictator giving, performed without the presence of the experimenter, have reported similar proportions of giving (Barmettler et al., 2012).

The above results have been explained in the terms of a “warm glow”—i.e., utility derived from the act of giving itself, regardless of its outcomes (Andreoni, 1990, 1993). Support for a quite literal interpretation of the warm glow hypothesis comes from the studies showing that giving money away to others produces positive feelings (Konow, 2010; Aknin et al., 2013). Although both 7-year-olds and 9-year-olds share their resources in a fair way, only the latter group feels better after doing this—suggesting that warm glow might require some experience to develop (Kogut, 2012). However, there is also some evidence for warm glow in children as young as two years (Aknin et al., 2012). Before this age children have already begun to engage in spontaneous helping behavior (Brownell and Carriger, 1990; Zahn-Waxler et al., 1992; Warneken and Tomasello, 2007; Liszkowski et al., 2008; Brownell et al., 2009; Bischof-Köhler, 2012) and are able to use model-free representations to guide behavior (Klossek et al., 2008), so it is plausible that warm glow effects could rely on the habitual system, but their developmental trajectory remains to be established. Results from experiments investigating warm glow bear striking similarity to those usually seen in the devaluation procedure—that is, persistence in performing an action because of its intrinsic value, despite diminished value of its outcome. Therefore one can speculate that they both in fact describe the same process. Consistently, on the neural level both habitual actions and warm glow-giving engage the ventral and dorsal striatum (Harbaugh et al., 2007).

Another feature shared by habits and some forms of prosocial behavior is automaticity, characterized by effortlessness and rapidness. One of most popular procedures used to study automaticity is working memory load, which is thought to impair the functioning of the goal-directed system and increase reliance on the habitual system (Otto et al., 2013). Schulz et al. (2014) used this manipulation in a series of mini-dictator games, in which participants had to make binary choices between arbitrarily defined equal and unequal divisions of money. They found that working memory load increased the proportion of fair choices. Importantly, this increase was present for all decisions, irrespective of the level of unfairness of the alternative, in sharp contrast to the control condition, where participants’ decisions were highly dependent on the degree of advantageous inequity—an effect that mirrors the insensitivity of the habitual system to the consequences of one’s actions. Consistently, other studies have found that working memory load also decreased the strategic tendency to defect near the end of the repeated prisoner’s dilemma, reflecting blindness of the habitual system to future (Duffy and Smith, 2014).

Conceptualizing some prosocial behaviors as habitual actions can also potentially explain variation in prosocial behavior. Individual differences in prosocial orientation might stem to some extent from varying levels of the automatization of other-regarding acts, emerging due to different personal experiences. In support for this claim, working memory load enhances prosocial behavior only for individuals with a prosocial orientation measured by questionnaires, but has an opposite effect on individuals with a proself orientation (Cornelissen et al., 2011)—consistent with the notion that prosocial behaviors of the first group might be more habitual and the second group more goal-directed. Studies using an ego-depletion procedure found similar results for proself oriented individuals, and slightly less consistent results for prosocial individuals (Balliet and Joireman, 2010; Halali et al., 2013).

Complementing these findings, some studies have shown that prosocial decisions are faster (Rand et al., 2012; Lotito et al., 2013; Rand et al., 2013) and are increased under time pressure, especially for subjects who do not have experience with anonymous one-shot economic games promoting self-interested acts (Cappelletti et al., 2011; Rand et al., 2012, 2014). However, it is important to note that some studies failed to replicate these results (Tinghög et al., 2013; Verkoeijen and Bouwmeester, 2014), and prosocial decisions concerning harm to others are slower than selfish decisions (Crockett et al., 2014).

We have shown many parallels between habits and prosocial acts on the behavioral level. Do habits and prosocial acts also engage similar neurocomputational mechanisms? Support for the applicability of the critic component of the actor-critic model in the context of prosocial behaviors comes from studies showing that outcomes of social interactions in economic games (Rilling et al., 2004; Zhu et al., 2012), violations of social norms (Klucharev et al., 2009; Xiang et al., 2013), signs of social approval (Jones et al., 2011) and even consequences of transferring money to charity (Kuss et al., 2013) are encoded in the form of reward prediction errors in the ventral striatum, in line with the possibility that this signal is used to update the expected value of other-regarding acts. Much less is known about the dorsal striatum and its role as an actor in this context, although some studies indeed found that activity of this brain part can be predictive of some other-regarding acts (Rilling et al., 2004; de Quervain et al., 2004; King-Casas et al., 2005; Harbaugh et al., 2007).

Separate lines of research focused on the influence of intuition, warm glow and habitual control in promoting prosocial behavior seem to converge in showing that other-regarding acts can be reinforced by experience, automated and have an intrinsic value. Future work will need to assess to what extent these disparate findings are actually characterizing the same process, vs. unique phenomena—an endeavor that can be facilitated by well-defined computational and neural characteristics of the habitual system. An important caveat is that automaticity and independence of responses from working memory are also features of the Pavlovian system, and therefore many of the above findings could be also attributed to Pavlovian control. To resolve this issue, future experiments will need to carefully control for current motivational states and experience with the given type of social interactions, as the habitual system should be insensitive to the former but sensitive to the latter. In the next section, we will discuss the potential contribution of a reflexive Pavlovian system that both complements and competes with the goal-directed and habitual systems for control of prosocial behavior.

### Pavlovian Prosocial Behavior

Recent advances in developmental psychology have shown that infants are probably closer to Rousseau’s noble savages than Locke’s moral blank slates, as they are armed from birth with mechanisms allowing them to evaluate moral acts and favor, in many cases, good over evil (Bloom, 2013). However, beyond judging other’s behavior, are infants also predisposed to behave prosocially? In this section we will review evidence suggesting that some other-regarding acts might be inborn and triggered by evolutionary old mechanisms embedded in the Pavlovian system.

First we consider the possibility that some prosocial tendencies expressed early in development might have a flavor of innate Pavlovian reflexes. There is ample evidence showing that children around the age of 15 months start to engage in sharing, cooperating and consoling (Brownell and Carriger, 1990; Zahn-Waxler et al., 1992; Warneken and Tomasello, 2007; Brownell et al., 2009; Bischof-Köhler, 2012). Helping can be observed even earlier, at the age of 12 months (Liszkowski et al., 2008). These behaviors could be driven by a goal-directed system and a desire to increase others’ welfare. However, children before the age of 24 months do not seem to choose actions based on the predicted value of their outcomes (Klossek et al., 2008; Kenward et al., 2009), suggesting they are unlikely to engage in prosocial behaviors due to valuing their consequences. Alternatively, early social experiences and interactions with parents could reinforce prosocial behaviors and promote formation of prosocial habits. However, parental encouragement does not increase helping (Warneken and Tomasello, 2013) and external rewards can even hinder it in 20-month-old infants (Warneken and Tomasello, 2008). The last possibility is that prosocial behaviors are driven by some inborn factors. In line with this, researchers have observed similar developmental patterns of sharing and cooperating in early childhood across different cultures (House et al., 2013), as well as examples of helping and consolation in different species, including apes (Warneken and Tomasello, 2006; Romero et al., 2010), rats (Bartal et al., 2011) and birds (Seed et al., 2007).

What innate mechanism could potentially drive prosocial behaviors? Affective empathy constitutes a likely candidate (de Waal, 2008; Bischof-Köhler, 2012). It develops on the basis of emotional contagion—i.e., the automatic matching between one’s own emotional state and the state of the perceived other (Preston and de Waal, 2002). Notably, emotional contagion is present from birth and also found in other mammals (Dondi et al., 1999; Langford et al., 2006; Nakashima et al., 2015). When children develop a self-other distinction around the age of 15 months, they also start to be aware that shared feelings originate from the state of the other person and are able to volitionally attend to it or not—an ability that constitutes an essence of affective empathy (Preston and de Waal, 2002; Bischof-Köhler, 2012). From the age of 18 months children are also able to infer the emotional states of others not only from emotional expressions but also from situational contexts (Vaish et al., 2009), implying that from early on we possess sophisticated capabilities of affective perspective taking.

Affective empathy has been associated with other-regarding acts in many studies. First, the occurrence of various prosocial behaviors correlates with the development of a self-other distinction and, in consequence, with the development of affective empathy in children (Brownell and Carriger, 1990; Zahn-Waxler et al., 1992; Bischof-Köhler, 2012). Second, self-reported measures of affective empathy correlate with various prosocial behaviors in adults (Eisenberg and Miller, 1987). Third, observing another’s suffering is a potent motivator of other-regarding acts: rats pull levers to terminate the distress of other rats (Bartal et al., 2011), monkeys refuse to pull a lever delivering food if it also delivers electric shocks to another monkey (Masserman et al., 1964), and humans are willing to swap places with a suffering person receiving shocks (Batson et al., 1987). Finally, impairment of affective empathy may play a causal role in antisocial behavior in psychopathy (Blair, 2005; Shamay-Tsoory et al., 2010).

Why would affective empathy promote prosocial behaviors? Feeling empathy towards a suffering individual is a source of a negative arousal and therefore other-regarding acts could be potentially driven by an instrumental motivation to eliminate it—either by bringing relief to someone or escaping from the source of distress (Cialdini et al., 1987). Consequently, habitual system should reinforce the action that lead to removal of aversive stimulus. However, humans exposed to other’s suffering are more willing to help, even when they can avoid the whole situation in an easy and costless way—which suggests that, at least in case of humans, empathy might trigger an approach rather than a withdrawal reaction (Batson et al., 1987; Stocks et al., 2009). This approach reaction is consistent with the empathy-altruism hypothesis, according to which feeling empathic concern for someone in need can evoke a genuine preference for the other’s well-being—a claim which has received solid support throughout the years (Batson et al., 1987; Batson, 2011). Importantly, dependence of this reaction on the state of empathic concern is inconsistent with the involvement of habitual system, as this system is insensitive to motivational states.

We speculate that the mechanism described by the empathy-altruism hypothesis has a Pavlovian character. More specifically, we propose that cues signaling harm or need, such as sad faces, may trigger an automatic urge to help, but only if a person is in the appropriate motivational state, that is, feels empathic concern for the other person. In line with this, 10 month old infants do not withdraw from victims of aggression, but instead show a preference for them, in comparison to both neutral objects and aggressors—an effect that might be interpreted as a rudimentary and perhaps inborn form of concern about the other’s well-being (Kanakogi et al., 2013).

Further support for the notion of a Pavlovian urge to help triggered by empathic concern comes from experiments demonstrating that inducing empathic concern can eclipse other goals and lead to maladaptive behaviors—just as in the case of the negative auto-maintenance procedure. For example, people make unfair decisions in favor of a person for whom they feel empathy, even when some other person is in greater need (Batson et al., 1995a); they unconditionally cooperate with an empathized target in the prisoner’s dilemma, even when the target has already defected (Batson and Moran, 1999; Batson and Ahmad, 2001); and they allocate more money to an empathized target, even at the cost of lower payouts for the whole group and damaging their own reputation (Batson et al., 1995b, 1999). Moreover, empathy induction in one context does not increase willingness to help the empathized target in other contexts—excluding the possibility that this procedure leads to generalized concern about other’s well-being (Dovidio et al., 1990). These examples demonstrate a Pavlovian-like inflexibility and specificity of the empathy-induced other-regarding reaction.

If this reaction is indeed Pavlovian, what could be its evolutionary origin? One proposition is that empathic concern stems from an over-generalization of the parental care instinct (Preston and de Waal, 2002; de Waal, 2008; Batson, 2010). Caretaking in mammals has a very strong reflexive component—as illustrated by a study in which both male and female virgin rats, with paralyzed voluntary muscle control, showed nursing behavior when exposed to unfamiliar pups (Stern, 1991). Furthermore, many animals have been observed to adopt unrelated orphans, suggesting that in some cases childlike features might be sufficient to evoke a parental care reflex and altruistic behaviors (Boesch et al., 2010). In line with this, it has been found that people are more likely to help and care for others possessing childlike facial and vocal characteristics, irrespective whether they are children or adults (Keating et al., 2003; Lishner et al., 2008; Glocker et al., 2009). It is possible that, due to some environmental pressures, a Pavlovian system evolved in humans to trigger caretaking reactions to a wider range of stimuli than only to infants. What makes this claim plausible is that humans show signs of alloparenting and cooperative breeding—that is, taking care of children that are not their own and are often genetically unrelated (Burkart et al., 2014). Crucially, cooperative breeding requires increased responsiveness and an attentional bias towards signals of need. These requirements may have predisposed us to feel empathic concern in a broad array of situations (Burkart and van Schaik, 2010). Consistently, across 15 species of primates, the extent of engagement in cooperative breeding is one of the best predictors of other-regarding preferences in social interactions with strangers (Burkart et al., 2014).

In addition to guiding prosocial behaviors directly through inborn reflexes, the Pavlovian system may modulate habitual or goal-directed other-regarding tendencies through Pavlovian-to-instrumental transfer (PIT). We assume that prosocial behaviors have an approach character, and as such can be invigorated by presence of appetitive cues and inhibited by aversive cues. Some preliminary evidence in support of this claim comes from studies measuring reaction times of prosocial decisions. In general, it has been found that other-regarding acts are faster than self-regarding acts in the context of rewards—an effect that was interpreted as evidence for the automaticity of such responses (Rand et al., 2012, 2013; Lotito et al., 2013). However, a recent study has shown that altruistic individuals make slower decisions when they decide for others in the context of punishments (Crockett et al., 2014), suggesting that the difference in reaction times between rewarding and punishing contexts might stem from Pavlovian invigoration and inhibition of instrumental approach reactions.

Aside from modulating the vigor of responding, could PIT also increase the tendency to act prosocially? We suggest that indeed prosocial dispositions could be enhanced by a range of Pavlovian cues triggering approach reactions towards people, either through evoking positive arousal or increasing expectation of positive outcomes—effects which could be also interpreted as changes in mood and inferences about outcomes of social interaction. Happy expressions and direct eye-gaze could be examples of such Pavlovian cues: two-day-old newborns look longer at happy faces, in comparison to fearful and neutral ones (Farroni et al., 2007), and also at faces making direct eye-contact with them, in comparison to the ones with averted gaze (Farroni et al., 2002). These same cues also increase prosocial behaviors later in life: smiling faces increase helping and cooperating in one-shot social interactions (Scharlemann et al., 2001; Guéguen and De Gail, 2003; Reed et al., 2012; Mussel et al., 2013); and pictures of eyes increase prosocial behaviors in anonymous dictator games and charitable donations in field experiments (Haley and Fessler, 2005; Rigdon et al., 2009; Powell et al., 2012; but see: Fehr and Schneider, 2010). As most of these studies focused on happy expressions and compared them to neutral expressions, future work will need to address the question if also other signs of experiencing emotions can work as Pavlovian cues.

Cues of familiarity and similarity might also enhance prosocial tendencies through PIT, as they also trigger reflexive approach reactions: newborns and infants prefer familiar faces (Barrile et al., 1999; Kelly et al., 2005) and 10 month olds prefer individuals with similar tastes to themselves (Mahajan and Wynn, 2012; Hamlin et al., 2013). Attraction towards familiar and similar others probably evolved as a heuristic for identifying and favoring kin—a highly beneficial ability from the perspective of spreading copies of one’s genes (Hamilton, 1964; Lieberman et al., 2007). However, these cues also enhance prosocial behaviors in many other situations. For example, seeing a picture or knowing a surname of the recipient in the dictator game increases willingness to share the endowment (Bohnet and Frey, 1999; Burnham, 2003; Charness and Gneezy, 2008); and membership in the same group (Ahmed, 2007; Halevy et al., 2012) or having similar facial features with another person (DeBruine, 2002; Krupp et al., 2008) promotes other-regarding acts in various economic games.

It could be argued, that aggression and urge to punish somebody are an approach reactions and therefore, according to the above account, should also be enhanced by appetitive cues. However, aggression and punishment can have a dual character: either prosocial, as in the case of punishments in the ultimatum game for violating social norms, or antisocial, as in the case of spite. We speculate that prosocial or antisocial nature of these actions provides a higher order context for the Pavlovian system. Consequently, we predict that appetitive cues will invigorate prosocial punishment and will inhibit antisocial punishment. As none of the studies so far has directly tested this hypothesis, future work will need to fill in this gap.

Other findings can also be re-interpreted through the lens of classical conditioning and PIT effects. Earlier we discussed the study by Peysakhovich and Rand (2013), in which repeated play of a prisoner’s dilemma in settings promoting defection increased a general tendency to act in a self-interested manner in other economic games. Involvement of the habitual system in the above findings might be questionable in light of the low generalizability of habits across contexts in other experiments using non-social stimuli (Krakauer et al., 2006; Hilario et al., 2012). An alternative explanation proposes that participants associated defecting anonymous players with a negative feeling through classical conditioning. This negative association could then stain subsequent interactions with other anonymous players in other games through PIT. To test this directly, future work will need to measure physiological reactions to anonymous players, while participants gradually acquire negative association. Supporting this idea, cooperation and defection in the prisoner’s dilemma has been shown to increase and decrease, respectively, the likeability of other player’s faces, as well as modulate amygdala responses to these faces in subsequent task (Singer et al., 2004).

In this section we have shown that many theories about the causes of prosocial behaviors can be re-interpreted in terms of Pavlovian reflexes and the mechanism of PIT. According to this view, the Pavlovian system can compete with other RLDM systems for behavioral control and trigger automatic prosocial behaviors in response to perceiving signals of need and feeling empathic concern for others. Alternatively, the Pavlovian system could interact with other RLDM systems by enhancing the likelihood and vigor of prosocial acts in the presence of stimuli evoking approach reactions towards other people.

## Discussion

In this review we summarized evidence showing how the RLDM framework can integrate diverse findings describing what motivates prosocial behaviors. We suggested that the goal-directed system, given sufficient time and cognitive resources, weighs the costs of prosocial behaviors against their benefits, and chooses the action that best serves one’s goals, whether they be to merely maintain a good reputation or to genuinely enhance the welfare of another. We also suggested that to appreciate some of the benefits of other-regarding acts, such as the possibility of reciprocity, agents must have a well-developed theory of mind and an ability to foresee the cumulative value of future actions—both of which seem to involve model-based computations.

Furthermore, we reviewed findings demonstrating that the habitual system encodes the consequences of social interactions in the form of prediction errors and uses these signals to update the expected value of actions. Repetition of prosocial acts, resulting in positive outcomes, gradually increases their expected value and can lead to the formation of prosocial habits, which are performed without regard to their consequences. We speculated that the expected value of actions on a subjective level might be experienced as a ‘warm glow’ (Andreoni, 1990), linking our proposition to the behavioral economics literature. We also suggested that the notion of prosocial habits shares many features of the social heuristics hypothesis (Rand et al., 2014), implying that the habitual system could be a possible neurocognitive mechanism explaining the expression of social heuristics.

Finally, we have posited that the Pavlovian system, in response to another’s distress cues, evokes an automatic approach response towards stimuli enhancing another’s well-being—even if that response brings negative consequences. We have also proposed that presence of appetitive and aversive stimuli can increase or decrease the vigor of prosocial reactions through the mechanism of Pavlovian-to-instrumental transfer. Pavlovian-to-instrumental transfer could also be responsible for the enhancing effects of familiarity, similarity, happy expressions and pictures of eyes on prosocial acts.

In addition to organizing a diverse set of findings on patterns of prosocial behavior, the RLDM framework also provides insight into possible sources of individual differences, developmental changes and interspecies variability in prosocial tendencies. Furthermore, by connecting behavioral economics, psychology, cognitive neuroscience, evolutionary biology and machine learning, this scheme opens new avenues of research at the boundaries of these disciplines.

However, explaining prosocial behavior within the RLDM framework is far from complete. There is an ongoing debate concerning the basic neural circuitry of the goal-directed, habitual and Pavlovian system, and researchers have only recently begun to uncover how these systems cooperate and compete with one another (Dolan and Dayan, 2013; Lee et al., 2014). Meanwhile, there is still relatively little work elucidating the neural substrates of prosocial behaviors, and almost none of this research has attempted to explain prosocial behaviors explicitly in terms of RLDM mechanisms. Future work will need to especially focus on the instances of prosocial behaviors which could be under the control of more than one system and utilize paradigms used in classical RLDM literature to disentangle influence of each of the three systems.

Here we mainly focused on the role of the DLPFC and striatum in motivating prosocial behaviors—the former being a crucial hub of model-based computations used for goal-directed behavior, and the latter responsible for the formation of habits and approach reactions towards stimuli. It is important to note that many of the studies cited in this review also reported the involvement of other neural circuits associated with the RLDM framework, such as the orbitofrontal cortex and the amygdala. However, their precise functional role in other-regarding decisions is less clear than the role of the DLPFC and striatum. Furthermore, it is known that brain regions involved in affective processing and social cognition, such as the anterior insula, anterior cingulate cortex, medial prefrontal cortex and temporo-parietal junction, also play a vital role in prosocial behaviors—although traditionally they are not considered to be a part of the RLDM neural circuitry (Singer et al., 2006; Hare et al., 2010; Morishima et al., 2012; Waytz et al., 2012; Smith et al., 2014). Following other authors, we suggest that information encoded by these regions serves as an input for the three decision-making systems used to predict the consequences of one’s actions in social situations and compute the values of different states of the world (Phelps et al., 2014; Ruff and Fehr, 2014).

Under the assumption that the three decision-making systems described here indeed govern prosocial behaviors, it is possible to generate a number of specific predictions that have yet to be tested. First, according to the RLDM framework, the goal-directed system might use heuristics to narrow down the range of considered scenarios, such as discarding action sequences which produce immediate and substantial negative outcomes—a process described as Pavlovian ‘pruning’ (Huys et al., 2012). Such a process could be responsible for selfish decisions in situations involving immediate personal costs, despite much greater potential social benefits. Therefore it is speculated that costly prosocial behaviors could be enhanced by situating the personal costs later in the action sequence. Second, irrelevant cues evoking approach and withdrawal reactions towards another person could potentially invigorate or inhibit prosocial tendencies towards this person, through the mechanism of PIT (Bray et al., 2008)—a feature that could be used, for example, in fund-raising advertisements. Third, the formation of habits requires in the training phase that the learner experience an instrumental contingency between responses and outcomes; in other words, the learner has to feel that given actions are associated with some positive value (Keramati et al., 2011). Actions that simultaneously bring counterbalanced appetitive and aversive consequences will have a net value close to zero and therefore will be immune to habitization. From this one could predict that costless other-regarding acts will be particularly prone to becoming habits, while prosocial behaviors requiring difficult trade-offs will probably stay under the control of the goal-directed system. Finally, it is well established in the RLDM literature that random schedules of reinforcements, that is reinforcements delivered at unpredictable intervals, lead to rapid habit formation (Derusso et al., 2010). Therefore one could speculate that uncertainty embedded in social interactions is particularly well-suited to automatize prosocial behaviors, as other-regarding acts are not always and not immediately rewarded. Knowledge of which conditions are the most effective in creating habits could potentially be used in designing interventions to promote prosocial tendencies.

We began this review by referring to a question about the true motivation behind prosocial behaviors. Perhaps not surprisingly, dissecting the mechanisms shaping other-regarding acts reveals a blend of altruistic and egoistic motives at their source. What is important, however, is that reinforcement learning mechanisms are able to transform egoistic motivations into prosocial behaviors, as in the case of prosocial habits formed on the basis of repetition of egoistically motivated other-regarding acts, and altruistic motivations into antisocial behaviors, as in the case of empathic concern for one person eclipsing the well-being of people in greater need. These insights, among others described here, demonstrate the potential for this line of research to help improve society.

## Conflict of Interest Statement

The authors declare that the research was conducted in the absence of any commercial or financial relationships that could be construed as a potential conflict of interest.
